# Programmed Cell-Death Mechanism Analysis Using Same-Cell, Multimode DNA and Proteoform Electrophoresis

**DOI:** 10.1021/acsmeasuresciau.1c00014

**Published:** 2021-08-17

**Authors:** Ana E. Gomez Martinez, Amy E. Herr

**Affiliations:** † Department of Bioengineering, University of California Berkeley, Berkeley, California 94720, United States; ‡ The University of California Berkeley and University of California San Francisco Graduate Program in Bioengineering, Berkeley, California 94720, United States; § Chan Zuckerberg Biohub, San Francisco, California 94158, United States

**Keywords:** Apoptosis, DNA fragmentation, PARP1, electrophoretic cytometry, polyacrylamide gel, proteoform expression, single-cell

## Abstract

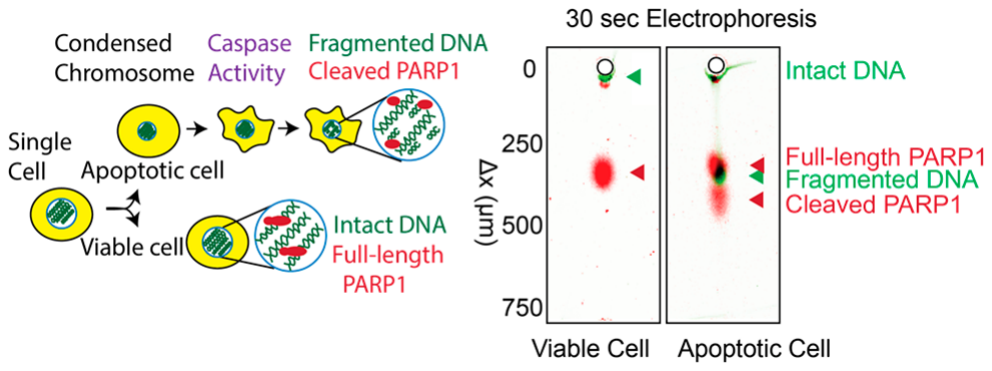

Gaining insight into the timing of cell apoptosis events requires single-cell-resolution measurements of cell viability. We explore the supposition that mechanism-based scrutiny of programmed cell death would benefit from same-cell analysis of both the DNA state (intact vs fragmented) and the protein states, specifically the full-length vs cleaved state of the DNA-repair protein PARP1, which is cleaved by caspase-3 during caspase-dependent apoptosis. To make this same-cell, multimode measurement, we introduce the single-cell electrophoresis-based viability and protein (SEVAP) assay. Using SEVAP, we (1) isolate human breast cancer SKBR3 cells in microwells molded in thin polyacrylamide gels, (2) electrophoretically separate protein molecular states and DNA molecular states—using differences in electrophoretic mobility—from each single-cell lysate, and (3) perform in-gel DNA staining and PARP1 immunoprobing. Performed in an open microfluidic device, SEVAP scrutinized hundreds to thousands of individual SKBR3 cells. In each single-cell lysate separation, SEVAP baseline-resolved fragmented DNA from intact DNA (*R*_s_ = 5.17) as well as cleaved PARP1 from full-length PARP1 (*R*_s_ = 0.66). Comparing apoptotic and viable cells showed statistically similar profiles (expression, mobility, peak width) of housekeeping protein β-tubulin (Mann–Whitney U test). Clustering and cross-correlation analysis of DNA migration and PARP1 migration identified nonapoptotic vs apoptotic cells. Clustering analysis further suggested that cleaved PARP1 is a suitable apoptosis marker for this system. SEVAP is an efficient, multimode, end-point assay designed to elucidate cell-to-cell heterogeneity in mechanism-specific signaling during programmed cell death.

## Introduction

Dysregulated apoptosis mechanisms contribute to neurodegenerative disorders,^1^ cancers,^1,2^ and chemotherapeutic resistance.^3^ Understanding cancer-cell-death avoidance and chemotherapy resistance to determine drug targets requires scrutiny of programmed cell-death pathways, including apoptosis.^3^ During apoptosis, the cellular membrane blebs, DNA condenses, the caspase cascade modifies proteins, and DNA is fragmented into ∼50 kb fragments by active DNases.^4^

Accurate temporal resolution of apoptotic events requires single-cell resolution^5^ due to the asynchronous nature of apoptosis.^2^ The gold standard measurement of apoptosis is morphological changes (membrane blebbing, nuclear fragmentation). Other common measurements are imaging- or flow-cytometry-based analyses of stains.^2^ These stains penetrate a compromised cell membrane (e.g., ethidium homodimer-1, propidium iodide, SYTOX Green) or detect externalized phosphatidylserine (Annexin V).^2^

Beyond DNA damage, cancer-cell survival is influenced by the expressed repertoire of proteins in their various forms (proteoforms). Proteoforms comprise the myriad of molecular forms of a protein that arise from genetic variation, alternatively spliced RNA, and post-translational modification.^6^ Protein post-translational modifications, including phosphorylation and cleavages, are prevalent in mechanisms of apoptosis.^7^ Bulk proteoform measurements have been made with standard Western blots to investigate the apoptosis pathways after dosing with specific drugs/chemicals.^8−10^

In the classical caspase-dependent apoptosis mechanism^2^ found in mammalian cells, active caspase-3/7 cleaves some or all of the DNA-repair protein PARP-1.^11,12^ The full-length 116 kDa PARP1 protein is cleaved into 89 and 24 kDa fragments during apoptosis.^12^ The caspase-3/7 activity leads to activation of caspase-dependent DNases and then the fragmentation of DNA,^13^ into 50 kbp fragments.^4^ Therefore, PARP1 is cleaved slightly upstream of DNA fragmentation during apoptosis, but both events occur in similar stages of apoptosis. Additionally, PARP1 may be cleaved by a caspase-independent nonapoptotic pathway (TGF-β induced in mice liver cell lines)^14^ or by caspase-7 during a nonapoptotic proinflammatory response.^15^

Detection of DNA damage has historically been accomplished through detection of fragmented DNA using electrophoretic analysis of lysate from individual cells. In single-cell gel electrophoresis, or the comet assay, individual cells are embedded in an agarose layer, cells are lysed, and the fragmented DNA is subjected to agarose electrophoresis.^16^ The intact versus fragmented DNA state is measured owing to differences in electrophoretic mobility as the DNA migrates through the agarose sieving matrix. The namesake “comet” refers to pattern generated by the overlapping of the DNA fragment bands, which can resemble the tail of a comet. The amount of DNA in the tail and tail length are used to determine the level of DNA damage.^17^ The comet assay is the gold standard for single-cell DNA damage measurements and is commonly used for fundamental research on DNA damage or repair and for genotoxicity testing of chemicals.^18^ Throughput of the comet assay has been increased by adopting microwells^19^ or microfluidic arrays^20^ for single-cell isolation.

The canonical comet assay does not resolve the various DNA fragments present in apoptotic cells, and the DNA damage measurement is saturated at several thousand breaks per cell, which is below the level of DNA fragmentation in a dead cell and does not measure cell viability.^21^ Fractionating the 50 kb DNA fragments from intact DNA should allow apoptotic cells to be identified and, when combined with analysis of proteoform expression, identify apoptosis mechanisms. Prepending electrophoresis to a single-cell-resolution immunoassay improves proteoform selectivity over an immunoassay alone (i.e., immunofluorescence, immunohistochemistry, flow and mass cytometry). Single-cell Western blotting is capable of assessing expression of specific proteoforms including truncated oncoproteins in cancer cells (e.g., HER2 and t-erbB2).^22^ Probed single-cell isoelectric focusing measures the expression of similar-mass isoforms with distinct isoelectric points.^23^

Consequently, we posit that a same-cell assay designed to measure both the DNA state and the state of a key apoptosis signaling protein such as PARP1 would be useful in opening inquiry into the programmed cell-death mechanism. We introduce the single-cell electrophoresis-based viability and protein (SEVAP) assay, capable of simultaneously identifying apoptosis and proteoform expression from thousands of cells per chip. This dual-mode measurement is achieved by fractionating the molecular forms of DNA and proteins in an open microfluidic device with a polyacrylamide gel (PAG) sieving matrix. A single-cell DNA mode and protein mode single-cell polyacrylamide gel electrophoresis assay were each optimized and then coupled to make a multimodal viability and proteoform measurement (SEVAP assay). The cleaved PARP1 was evaluated as a spontaneous apoptosis marker in SKBR3 cells. In the future, the high-throughput and simultaneous viability and protein expression measurement from individual cells may elucidate apoptosis mechanisms or chemotherapeutic resistance mechanisms.

## Experimental Section

### Chemicals

A solution of 30% (w/v) (29:1) acrylamide/bis acrylamide (A3574), *N*,*N*,*N*′,*N*′-tetramethylethylenediamine (TEMED, T9281), and ammonium persulfate (APS, A3678) was purchased from Sigma-Aldrich. *N*-[3-[(3-Benzoylphenyl)-formamido]propyl] methacrylamide (BPMAC) was synthesized by PharmAgra Laboratories. Dual-lysis/electrophoresis buffer is composed of 0.5× Tris-glycine from Bio-Rad (1610734), 0.5% sodium dodecyl sulfate (SDS) from Sigma-Aldrich (L3771), 0.25% sodium deoxycholate from Sigma-Aldrich (D6750), and 0.1% Triton X-100 from Sigma-Aldrich (X100–100 ML). 10 000× SYBR Green I (S7563) was purchased from Invitrogen. GeneRuler High Range DNA ladder (48 502, 24 508, 20 555, 17 000, 15 258, 13 825, 12 119, 10 171 bp) was purchased from Thermo Fisher Scientific (SM1351). Tris buffered saline with Tween 20 (TBST 10×) was purchased from Cell Signaling Technology (9997S). 10× Tris/borate/EDTA buffers (TBE) were purchased from Invitrogen (AM9863).

### Cell Culture

SKBR3 cells were cultured in DMEM, high-glucose, GlutaMAX Supplement (10566–016) with 1% penicillin/streptomycin (15140122, Life Technologies) and BenchMark FBS (100–106, Gemini Bio-Products). SKBR3 cells were authenticated by short tandem repeat analysis and tested negative for mycoplasma (Supporting Information).

### SU8 Wafer and PAG Fabrication

SU8 wafer was fabricated using photolithography as previously reported.^24^ The array was 44 μm high, the separation lane was 1 mm, the transverse distance between microwells was 400 μm, and the microwell diameter was 32 μm. All PAGs were 7%T (3.33%C), 3 mM BPMAC, 0.08% TEMED, and 0.08% APS, 1× Tris-glycine.

### DNA Ladder Electrophoresis

Ladder was diluted according to manufacturer directions and pipetted over the 7%T (3.33%C) PAG. Electrophoresis was 15 s in 1× TBE.

### SEVAP

**Figure 1:**
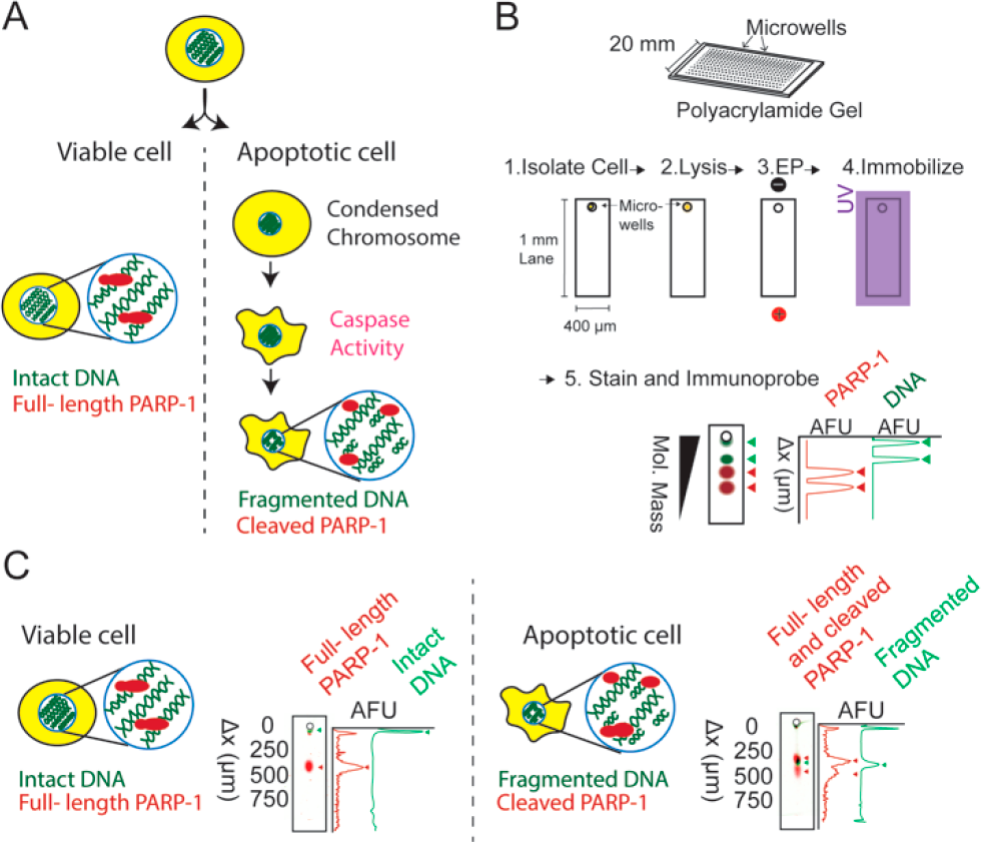
Microseparation simultaneously measures DNA fragmentation and protein expression in single cells. (A) Measuring PARP1 states (full-length vs cleaved) and DNA states (intact vs fragmented) in the same cell will help to investigate the programmed cell-death mechanism. In cells undergoing caspase-dependent apoptosis, dsDNA is fragmented, and PARP1 is cleaved. (B) SEVAP, a microarray open microfluidic device, isolates single cells and performs electrophoretic cytometry to analyze the DNA migration and proteoform expression. The PAG pore size, time of PAGE, and electric field are selected to analyze both DNA and proteoforms. (C) PARP1 states and DNA states are measured simultaneously in an end-point micrograph to analyze the proteoform expression in live and apoptotic cells.

Single cells were isolated in microwell arrays in polyacrylamide gels and lysed as previously reported.^24^ The microwells were 32 μm in diameter and placed 400 μm apart with 1 mm separation axes. The PAG is 44 μm thick. After polyacrylamide gel electrophoresis (PAGE) at 40 V/cm, proteins were immobilized by benzophenone (BP), which is covalently incorporated into the PAG and is activated by UV. Then, double-stranded (dsDNA) was stained with 1× SYBR Green I, and β-tubulin or PARP1 was immunoprobed. Proteins were immobilized by UV-activated benzophenone (Lightningcure LC5, Hamamatsu, 100% power, 45 s). 1× SYBR Green I (maximum excitation at 497 nm and emission at 520 nm) in diH_2_O stained the SKBR3 DNA and the high-range DNA ladder (16.7 ng/μL) (Figure 1B).

### Immunoprobing

Anti-β-tubulin (55 kDa) polyclonal rabbit antibody (diluted to 0.1 mg/mL in 2% BSA/1× TBST) was purchased from Abcam (ab6046), and the antirabbit antibody labeled with Alexa 647 (diluted to 0.1 mg/mL in 2% BSA/1× TBST) (A31573), with an excitation max of 650 nm and emission max of 665 nm, was purchased from Invitrogen. For each antibody, the 44 μm thick gel was probed for 1 h and washed for 2 h on a rotator with 1× TBST buffer exchange every 30 min.

Anti-PARP1 (cleaved and full) N-terminus monoclonal mouse antibody (Proteintech, Catalog # 66520–1-IG), was diluted 1:5 in 2% BSA/1× TBST and incubated for 2 h. The secondary antibody is donkey antimouse labeled with Alexa 594 (A21203, Invitrogen), with an excitation max of 590 nm and emission max of 617 nm, and was diluted 1:20 in 2% BSA/1× TBST and incubated for 1 h.

### Imaging

Fluorescence was imaged with 5 μm/pixel spatial resolution using a GenePix 4300/4400 Microarray Scanner from Molecular Devices (San Jose, CA). DNA ladder was imaged with a 10× magnification objective (Olympus UPlanFLN, NA 0.45, Tokyo, Japan). The Olympus IX71 inverted fluorescence microscope was connected to an Andor iXon+ EMCCD camera, an ASI motorized stage, and a shuttered-mercury-lamp light source (X-cite, Lumen Dynamics, Mississauga, Canada).

### Image Analysis

The micrographs were analyzed using custom scripts, as previously described,^24^ in MABLAB R2018b. Briefly, 400 μm by 1 mm regions of interest were created. Structures store the arbitrary fluorescence units (AFU) vertical line average profiles for DNA and proteins and the Gaussian fits to these profiles. Peak widths are the 4σ of the Gaussian fits from the intensity profiles (4σ fits 95% of the distribution), and the migration distances, or peak locations, are the center of the same Gaussian fits.

## Results and Discussion

For the multimode single-cell assay, we start by developing a single-cell proteoform separation mode to distinguish full-length versus cleaved PARP1. Next, we use knowledge acquired from that most-stringent molecular analysis (PARP1 forms) to design and develop the less-stringent, coarse DNA fractionation polyacrylamide gel electrophoresis (PAGE) mode. This latter mode is designed to distinguish intact versus fragmented DNA from one starting cell. Finally, we integrate the two modules such that both DNA and PARP1 states can be assessed from the same starting cell.

### Separation of PARP1 Proteoform States

We anticipate that distinguishing full-length from cleaved PARP1 will require more resolving power than fractionating intact versus fragmented DNA. As such, we first developed the protein immunoblot mode of the multimode SEVAP assay to resolve and measure full-length (116 kDa) and cleaved (89 kDa) PARP1 with the same antibody probe, an antibody against the N-terminus of PARP1. Cleaved PARP1 indicates caspase activity and caspase-dependent apoptosis^2^ (Figure 1A). SKBR3 cells are expected to express primarily full-length PARP1 (116 kDa), primarily cleaved PARP1 (89 kDa), or a combination of full-length and cleaved PARP1. The PARP1 signal readout is a fluorescence immunoassay (Figure 1B). When integrated, the DNA signal readout will also be fluorescence owing to the use of DNA staining.

To separate the 116 and 89 kDa PARP1 forms, we selected PAGE conditions (i.e., PAG pore size, time of PAGE, and applied electric field) (Figure 1C) to achieve a separation resolution, *R*_s_, of ∼1. The *R*_s_ increases with an increasing difference in protein migration distance or decreasing peak widths.^25^ The measured mobility was 3.18 × 10^–5^ cm^2^/(V s) ± 4.98 × 10^–7^ for the 116 kDa, or full-length, PARP1 and 4.03 × 10^–5^ ± 1.13 × 10^–6^ cm^2^/(V s) for the 89 kDa, or cleaved, PARP1 (mean migration distance for full-length PARP1 is 381 ± 6.0 μm and for cleaved PARP1 is 483 ± 13.6 μm with *n* = 5 lanes). The measured peak width for full-length PARP1 is 123 ± 5.5 μm and for cleaved PARP1 the peak width is 192 ± 32 μm. The measured *R*_s_ was 0.66 ± 0.14 after 30 s of PAGE. The PARP1 forms of interest have a 23% mass difference and were resolved in a 7%T PAG. PAGE can separate proteins by molecular mass, and both the full-length and cleaved PARP1 are selectively measured with the same antibody probe.

### Fractionation of DNA States

We next sought to develop the DNA mode of the SEVAP assay. As mentioned earlier, during caspase-dependent apoptosis, active DNases fragment DNA into 50 kb with some of the DNA remaining intact.^4^ Here, we seek to fractionate fragmented DNA (50 kb) from intact DNA to detect the apoptosis hallmark (50 kb fragments) and not measure the degree of DNA fragmentation as is done with the comet assay.

To achieve this coarse fractionation of the two DNA subpopulations—intact versus fragmented—we hypothesize that PAGE will provide the low-resolving power required. This underlying hypothesis is important, as the assertion forms the linchpin for the same-cell dual-mode SEVAP assay. If the coarse fractionation functionality is possible, SEVAP can be designed to integrate same-cell DNA and PARP1 molecular state measurements using the same PAGE conditions.

The small pore size of PAG (pore size is estimated below 10 nm^26^) results in DNA molecular reptation with stretching, where a biomolecule stretches under an applied electric field with a snake-like migration. In this reptation with stretching regime, the mobility vs molecular-mass curve plateaus for high-molecular-mass molecules.^27^ This behavior in the reptation with stretching regime leads to low-resolution separations of DNA fragments (10s kb) from each other in PAG. Increasing the applied electric field from the standard 1 V/cm for a comet assay^21^ further exacerbates this reptation with stretching and the mobility plateau. We hypothesize that at this high applied electric field, the 50 kb fragments will electrokinetically inject and electromigrate by reptation through the PAG, while intact DNA will remain in the microwell. In this way, we design the PAG sieving matrix to fractionate the fragmented from the intact DNA by acting as a size-exclusion filter. To our knowledge, the electrophoretic mobility of the DNA state that indicates apoptosis (i.e., 50 kb fragments) has not been reported in PAG at a high electric field (40 V/cm compared to 1 V/cm for DNA analysis).

**Figure 2:**
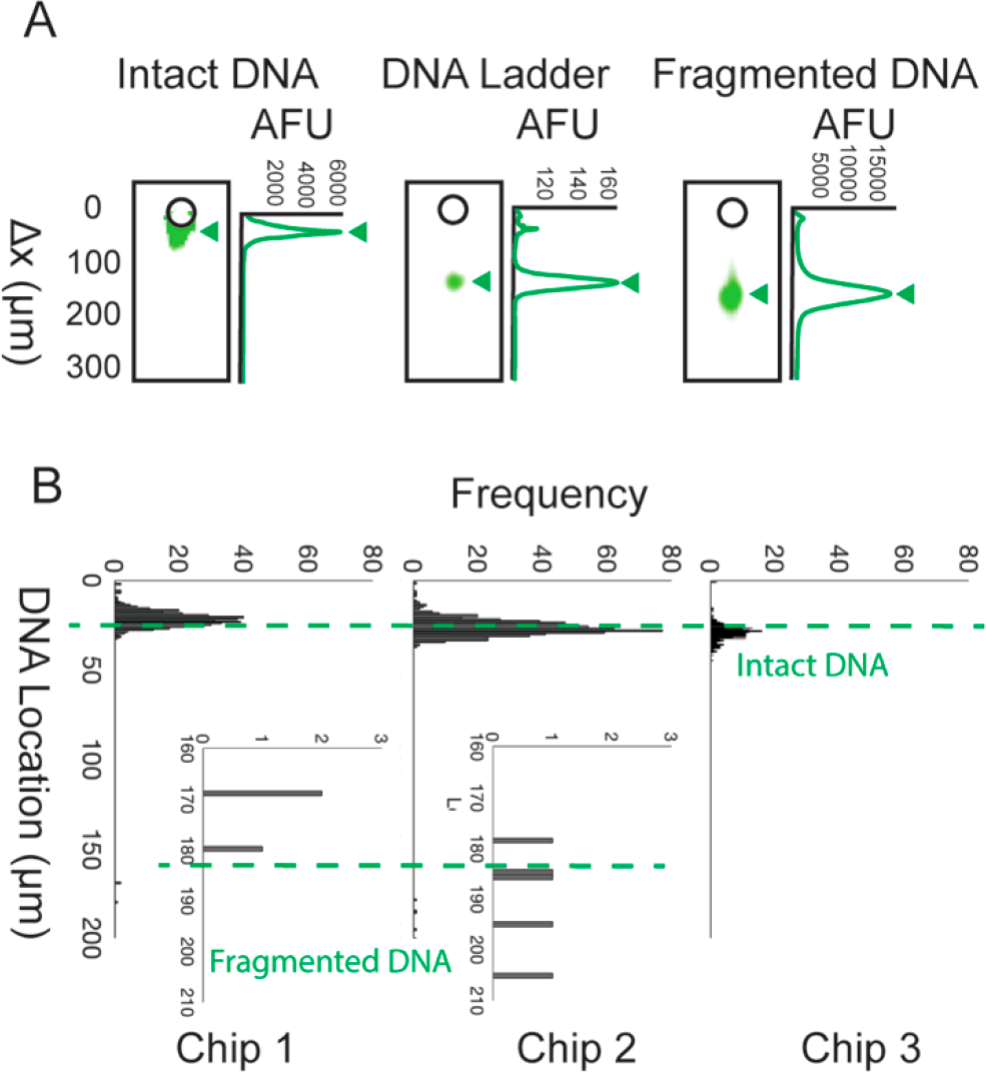
Viable and apoptotic SKBR3 cells are identified based on DNA migration repeatably via SEVAP. (A) The DNA ladder migrated 136 ± 3.3 μm (*n* = 7 lanes), and fragmented DNA migrated substantially similarily, while intact DNA remained near the microwell region. (B) Across three chips, the fragmented DNA migrated 183 ± 12.5 μm in PAG, while the intact DNA migrated 25.8 ± 5.34 μm from the center of the microwell (chip 1: *n* = 401 cells and *n* = 3 cells contain fragmentation DNA, chip 2: *n* = 618 cells and *n* = 5 cells contain fragmentation DNA, chip 3: *n* = 375 cells and no cells contain fragmented DNA at this low sample number). There was no overlap in the distribution of the migration distance of fragmented DNA and intact DNA.

We sought to study the electromigration of a high-molecular-mass DNA ladder to determine if coarse detection of apoptotic DNA fragments (i.e., fractionating intact DNA and DNA fragments) by PAGE is feasible. The size of the DNA ladder components is expected to be similar to that of the DNA fragments found in apoptotic cells. We expect that the components of the DNA ladder will not resolve from each other. Importantly, the DNA mode of the SEVAP assay need not resolve each of the DNA fragments, but must simply resolve the intact from fragmented populations. First, to determine the expected electromigration behavior of 50 kb DNA fragments under PAGE conditions suitable for protein separation (7%T, *E* = 40 V/cm, 15 s of PAGE), a high-molecular-mass DNA ladder was introduced into the microwells and analyzed (Figure 2A). This ladder migrated 136 ± 3.3 μm (μ = 2.27 × 10^–5^ ± 5.5 × 10^–7^ cm^2^/(V s); *N* = 7 lanes). As expected, the DNA ladder migrated as a single band, showing that (1) the DNA is in the reptation regime and (2) fractionation of 50 kb DNA fragments from intact DNA can be accomplished under the same separation conditions needed to resolve full-length from cleaved PARP1 protein states.

In order to measure apoptosis with the DNA mode of SEVAP, we aim to characterize the migration of DNA from single cells and categorize this DNA as fragmented or intact. Assuming a Gaussian distribution and based on the measured DNA ladder migration, 99.85% of the DNA ladder species are expected to migrate >126 μm after 15 s of PAGE. Therefore, single-cell DNA with a migration distance >126 μm is categorized as fragmented (Figure 2A), under the SEVAP separation conditions described. Based on this categorization, the average migration distance of the fragmented DNA from SKBR3 cells was 183 ± 12.5 μm (*n* = 8 cells) (Figure 2B). The mobility of this fragmented DNA in PAG is μ = 3.06 × 10^–5^ ± 2.08 × 10^–6^ cm^2^/(V s). Assuming a Gaussian distribution, 99.7% of the fragmented DNA from SKBR3 cells is expected to migrate between 145 and 220 μm. The dual-lysis/electrophoresis buffer has a higher conductivity than the TBE buffer, so greater Joule heating and a higher migration distance are expected from the cellular DNA in lysis/electrophoresis buffer compared to the migration distance of the ladder in 1× TBE. Intact DNA (>50 kbp) is size-excluded from the separation gel, so that large DNA remains in the microwell with a peak location at 25.8 ± 5.34 μm (*n* = 1386) from the center of the 32 μm diameter well (Figure 2A). Therefore, 99.7% of the intact DNA migrated between 9.78 and 41.8 μm (Figure 2B). The *R*_s_ between the intact and fragmented DNA was calculated based on the peak location of the intact and fragmented DNA in different lanes and was 3.37 (*n* = 8 fragmented, *n* = 1386 intact). The polyacrylamide acts as a cutoff filter, preventing migration of intact DNA strands. The fragmented DNA and intact DNA have nonoverlapping distribution within chips or across chips (Figure 2B). This shows that DNA fragments and intact DNA (i.e., DNA larger than 50 kb) are resolvable in the PAG sieving matrix and under PAGE conditions that are suitable for separating full-length and cleaved PARP1 (7%T, *E* = 40 V/cm).

We assessed the spontaneous apoptosis rate with the DNA mode of the SEVAP assay. SKBR3 cells were incubated in PBS for 30 min prior to SEVAP lysis and analysis. The SKBR3 cells were untreated, meaning no cytotoxins, chemotherapeutics, or stimuli were delivered to cells. Previously, the spontaneous apoptosis rate for SKBR3 cells cultured in RPMI 1640 medium over 24 h was 15 ± 6%.^8^ Here, cells with DNA that migrated greater than 126 μm, the minimum migration distance of the DNA ladder, were classified as apoptotic. We observed an apoptosis rate of 0.6% (i.e., 8 cells exhibited fragmented DNA out of 1394 cells; *n* = 3 chips). Assuming a constant apoptosis rate, the previously reported apoptosis rate is 0.31% per half hour with a distribution of 0.0–0.81%. The observed average SKBR3 cell apoptosis rate is within range of the expected apoptosis rate from literature. This concordance between our measured spontaneous apoptosis rate and rates reported in the literature suggests that SEVAP reports a DNA electrophoretic mobility that accurately identifies cell viability, in this novel PAG format.

### Same-Cell DNA Fragmentation and Protein Expression Measurements Are High-Throughput

We aimed to design SEVAP as a high-throughput assay capable of measuring DNA state and protein expression. To accomplish the high-throughput cytometry needed to detect rare apoptotic cells, we used an open microfluidic design. High-throughput is needed when you consider that 965 untreated SKBR3 cells are expected to be required to have a 95% confidence in identifying one apoptotic cell, given the 0.31% per half hour apoptosis rate. The open microfluidic SEVAP device contains 3150 microwells (18 rows, 175 columns, each abutting a 1 mm long separation lane, as is sufficient to resolve the PARP1 states [full-length vs cleaved] and to resolve the DNA states [fragmented vs intact]). This design allowed for thousands of microwells and lanes on a single chip. The SEVAP device reported the DNA states and β-tubulin expression of 1394 SKBR3 cells (*n* = 3 chips) after an 8 h assay (2 h of hands-on time). Most cells (1386 cells) contained DNA that was sequestered to the microwell. Eight cells contained fragmented DNA that electromigrated in the PAG (apoptosis rate is 0.6%). The small population of apoptotic cells was detected in a single SEVAP chip due to the high-throughput of the single-cell assay.

**Figure 3:**
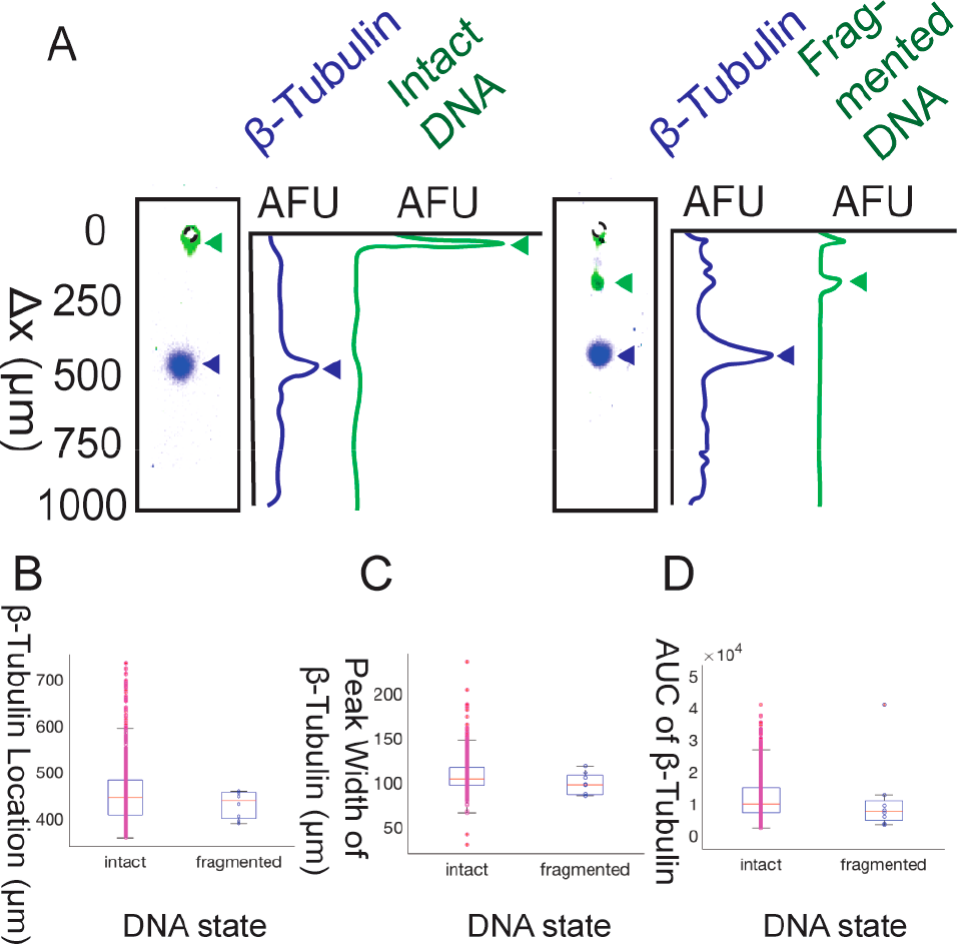
SEVAP is a high-throughput single-cell assay that measures two modes (intact vs fragmented DNA and proteoform expression) independently as shown by analyzing β-tubulin migration, peak width, and expression. (A) In the individual combined intensity profile of DNA and β-tubulin, DNA was contained near the microwell, or DNA electromigrated into the PAG. β-Tubulin is a control to determine if technical variation affects DNA mobility and if DNA location affects dispersion (*n* = 1394 cells). (B) The protein peak location of β-tubulin is similar between cells with intact and fragmented DNA*. (C) The β-tubulin peak width is similar between cells with intact and fragmented DNA. (D) The β-tubulin expression, or AUC, is similar between cells with intact and fragmented DNA. * Fragmented DNA have a peak location above 126 μm (the minimum migration distance of the 50 kb DNA ladder).

In order to exclude the possibility that the detection of high-mobility DNA was due to technical variation in SEVAP, we made multimodal measurements of the housekeeping protein β-tubulin (expression and mobility) and the DNA state of individual SKBR3 cells (Figure 3A). The average β-tubulin (55 kDa) migration is 455 ± 65.0 μm and the mobility is μ = 7.59 × 10^–5^ ± 1.08 × 10^–5^ cm^2^/(V s) (*n* = 1394 cells). The average coefficient of variance (CV) for β-tubulin peak location was 14.3% across three chips (chip 1: CV = 4.86%, *n* = 401 cells; chip 2: CV = 9.65%, *n* = 618 cells; chip 3: CV = 12.1%, *n* = 375 cells). There is no statistical difference in β-tubulin mobility between cells with fragmented and intact DNA (*n* = 1394 cells, Mann–Whitney U test) (Figure 3B). Comparison of DNA migration to that of the housekeeping protein supports the conclusion that the high mobility of the DNA is attributable to the DNA fragmentation rather than technical variation of the SEVAP, since this variation would be expected to influence β-tubulin mobility along with DNA mobility.

Furthermore, to exclude the possibility that a mass of DNA immobilized in the sieving gel might add sample dispersion to a protein peak, we analyzed the peak width for β-tubulin in lanes with fragmented versus intact DNA. If dispersion is influenced by DNA entrapment, then the peak width measurement could be affected by the DNA state. The mean peak width of β-tubulin in lanes with fragmented DNA was 99.1 ± 12.3 μm (*n* = 8), and the mean peak width of β-tubulin in lanes with intact DNA was 108.7 ± 16.6 μm (*n* = 1386) (Figure 3C). There was no statistical difference between these peak widths (Mann–Whitney U test). Therefore, the state of the DNA is not affecting protein dispersion.

We sought to analyze how β-tubulin compared in apoptotic and live cells. The β-tubulin expression is measured by AUC of the intensity profile. The β-tubulin is expressed statistically similarly (Mann–Whitney U test) in cells with fragmented and intact DNA (Figure 3D). The mean AUC of the β-tubulin in cells with intact DNA was 1.22 × 10^4^ ± 1.29 × 10^4^ AFU (*n* = 1386), and the mean AUC of the β-tubulin in cells with fragmented DNA was 1.16 × 10^4^ ± 1.23 × 10^4^ (*n* = 8). This invariant β-tubulin expression and molecular mass may be due to the required maintenance of the cytoskeleton during the apoptosis process, meaning no cytoskeletal degradation or protein downregulation takes place.

### Simultaneous Viability and PARP1 (Full-Length Vs Cleaved) Expression Measurements

As mentioned, PARP1 is cleaved by caspase activity and is an indicator of apoptosis.^7^ Full-length PARP1 (116 kDa) is cleaved during caspase-dependent apoptosis into 89 and 24 kDa. However, PARP1 cleavage may have other physiological functions.^1^ In first scrutinizing the protein mode of the SEVAP assay, we observed the full-length and cleaved PARP1 as detectable with the same N-terminus targeting antibody probe. SEVAP resolved the full-length (116 kDa) and cleaved PARP1(89 kDa) form by molecular mass in the sieving matrix (7%T PAG, 30 s PAGE, electric field was 40 V/cm). In the DNA mode of the SEVAP assay, we observed DNA states (intact vs fragments) resolved after 15 s of PAGE. Therefore, a 30 s PAGE resolved fragmented DNA from intact DNA and the full-length PARP1 from cleaved PARP1 from the same single cell. Since both fragmented DNA and cleaved PARP1 are present in caspase-dependent apoptosis, and caspase-dependent apoptosis is common, agreement between the two molecular markers is expected in a spontaneously apoptotic SKBR3 cell.

**Figure 4:**
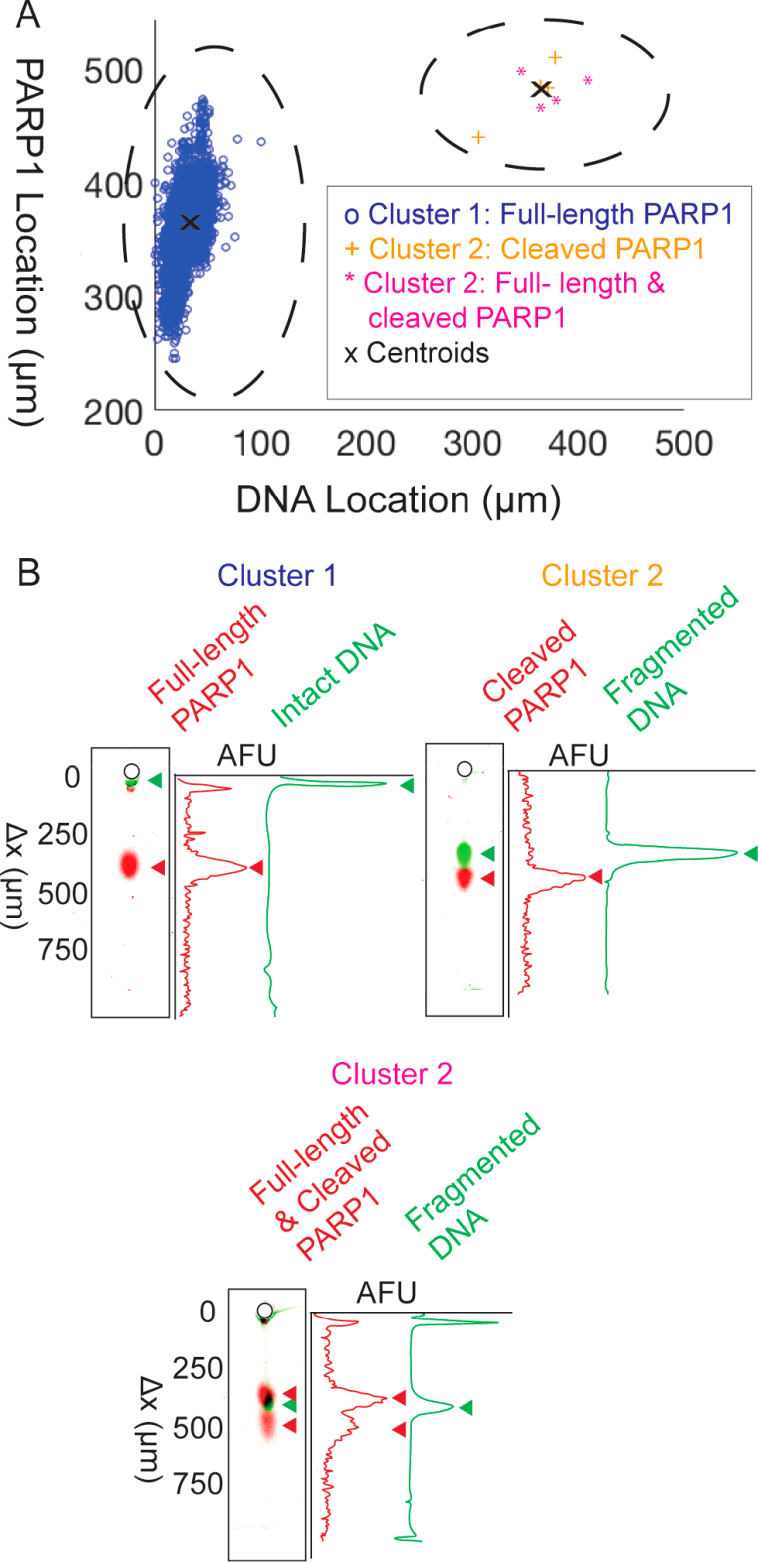
Clustering of PARP1 and DNA migration distance showed a live and apoptotic population, were cleaved PARP1 is present in only apoptotic cells (cells with fragmented DNA). (A) Cluster 1 contains live cells with intact DNA and only full-length PARP1 (*n* = 4507cells). Cluster 2 contained apoptotic cells with fragmented DNA and either some or only cleaved PARP1 (*n* = 8 cells). (B) Intensity profiles and micrographs of lanes show the populations and subpopulations in each cluster. Cluster 1 profiles were of live cells and contained full-length PARP1. Cluster 2 has one subpopulation (*n* = 4) of apoptotic cells containing cleaved PARP1. Cluster 2 has a second subpopulation (*n* = 4) of apoptotic cells containing both full-length and cleaved PARP1 in the same cell.

To evaluate if PARP1 is a spontaneous apoptosis marker for the SKBR3 cell line, we cross-correlated the presence of the two molecular markers, DNA and PARP1, in cells not exposed to any chemotherapeutic/stress agent. For this analysis, the PARP1 migration distance for cells with both forms of PARP1 was determined using the peak with the larger migration distance, because this peak indicates the expression of the cleaved PARP1. We clustered cells based on the PARP1 location and DNA location with a Gaussian mixture model and identified two clusters, the live and apoptotic cell clusters (Figure 4A). The mean migration distance of DNA for Cluster 1 was 33.1 ± 12 μm, and the mean migration distance of PARP1 for this cluster was 366 ± 33 μm. In Cluster 2, the mean migration distance of DNA migration was 365 ± 30 μm, and a mean migration distance of PARP1 was 481 ± 22 μm. Differences in the migration distances for PARP1 between these clusters are statistically significant (Mann–Whitney U test). Therefore, cells with fragmented DNA contained cleaved PARP1, and cells with intact DNA contained full-length PARP1. The PARP1 *R*_s_ is 0.78 (*n* = 8 cleaved PARP1, *n* = 4507 full-length PARP1), and the DNA *R*_s_ is 5.17 (*n* = 8 fragmented DNA, *n* = 4507 intact DNA). PARP1 is cleaved (89 and 24 kDa) by caspase during apoptosis and does not apply to other cell-death pathways such as necrosis. Apoptosis and necrosis have different morphological and molecular characteristics. In necrotic cells, DNA is fragmentated randomly,^28^ and PARP1 fragments may vary between 42 to 116 kDa.^29^ We observed discretely fragmented DNA and discretely cleaved PARP1, indicating these cells are apoptotic.

Three main cases of PARP1 states and DNA states were observed (Figure 4B). All viable cells, or cells in Cluster 1, contained intact DNA and full-length PARP1. All apoptotic cells express either both full-length and cleaved or only cleaved PARP1. Four apoptotic cells expressed only cleaved PARP1, and four other apoptotic cells express both full-length and cleaved PARP1. The migration distances measured by SEVAP demonstrate that spontaneously apoptotic SKBR3 cells express cleaved PARP1, and viable cells express only full-length PARP1.

## Conclusion

Here, the SEVAP assay reports proteoform expression and simultaneously measures viability from single cells. These simultaneous measurements remove any time lag between cell staining/imaging and protein separation, which may change the cell states. The capture of a single-time-point proteoform expression and viability for hundreds of cells can be leveraged for apoptosis investigation, which require precise timing and analysis of single cells. The relationship between fragmentation and DNA electrophoretic mobility is the basis for fractionating DNA by size and identifying apoptotic cells in polyacrylamide. A high-molecular-mass DNA ladder validated the migration of 50 kb fragmented DNA in PAG. Loading control protein, β-tubulin, showed no relationship to DNA fragmentation as expected. PARP1 was evaluated as a spontaneous apoptosis marker by correlation of the PARP1 proteoform expression to the DNA fragmentation in apoptotic cells.

Future work can lead to measuring and distinguish these cell-death pathways. After electrophoresis, DNA from necrotic cells appears as long smears, and DNA from apoptotic cell appears as bands. The presence of a single DNA band and of a maximum of two PARP1 bands indicate that the nonviable cells identified are apoptotic. Integrating knowledge about DNA fragmentation patterns through different death pathways and immunoprobing for proteins that are specific to death pathways can allow for more specific cell-death categorization (e.g., apoptosis vs necrosis). This future work could give more insight into the different cell-death pathway mechanisms. Measuring viability and proteoform expression simultaneously may be of special interest for analyzing protein cleaving, which is prevalent in the apoptosis process. The single-cell resolution measurement may allow for the timing of apoptosis events to be further studied.

SEVAP can be used to exclude an apoptotic population from data sets or to compare viable and apoptotic cell proteoform expression, using a single-step readout for hundreds to thousands of single cells. SEVAP may measure viability of cells in a heterogeneous samples such as tumors. Apoptosis mechanisms or chemotherapeutic resistance mechanisms may be studies with single-cell resolution. The multimode SEVAP assay can assist with investigating apoptosis mechanisms of new therapeutics to better understand how to reduce the risk of chemotherapeutic resistance.

## Supporting Information Available

Mycoplasma testing and cell line short tandem repeat (STR) report (PDF)
