# Supplementary material for: Programmed Cell-Death Mechanism Analysis Using Same-Cell, Multimode DNA and Proteoform Electrophoresis
Source: ACS Meas Sci Au. 2021 Aug 17;1(3):139–46. doi: 10.1021/acsmeasuresciau.1c00014 (PMC8679084; doi:10.1021/acsmeasuresciau.1c00014)
Supplement: Supplementary file 1 — tg1c00014_si_001.pdf [file tg1c00014_si_001.pdf]

Programmed Cell-Death Mechanism Analysis Using Same-Cell, Multimode DNA and Proteoform Electrophoresis

Ana E. Gomez Martinez[1, 2] and Amy E. Herr*[1, 2, 3].

[1]Department of Bioengineering, University of California Berkeley, Berkeley, California, 94720, USA. [2]The University of California Berkeley and University of California San Francisco Graduate Program in Bioengineering, Berkeley, California, 94720, USA. [3]Chan Zuckerberg Biohub, San Francisco, California, 94158, USA.

*aeh@berkeley.edu

Supporting Information

Mycoplasma testing and Cell Line Short Tandem Repeat (STR) Report are from 2019. Experiments were performed between July 2019 and October 2020.

University of California, Berkeley
Cell Culture Facility

Certificate of Analysis
Cell Line Short Tandem Repeat (STR) Report
Sample name: SKBR3
Date received: 10/30/19
Client: Alisha Geldert-UCB

**LOCUS**	**ALLELE SIZE**
THO1	8,9
D5S818	9,12
D13S317	11,12
D7S820	9,12
D16S539	9
CSF1PO	12
AMEL	X
vWA	17
TPOX	8,11

Results indicate the allele(s) detected at each locus tested. Each allele represents the number of short tandem repeats present at that locus. Generally, a DNA profile uniquely identifies an individual cell line. However, some cell lines may exhibit genomic instability over time leading to slight changes in the DNA profile.

**IDENTIFIED CELL TYPE(S)**	**PERCENT MATCH**
SK-BR-3	100%

Based on STR analysis, the results indicate a high probability (80% or higher) match with the cell line #1 listed. Multiple cell lines with ≥80% match are considered to be derived from common ancestry. Electropherograms are available upon request.

Disclaimer: While every reasonable effort has been made to assure the accuracy of these data, no warranty, express or implied, is made by this facility.

Alison Killilea

Alison N. Killilea, Ph.D. | Bioscience Facility Manager

University of California, Berkeley
Cell Culture Facility

Certificate of Analysis
Mycoplasma Testing
Sample names: BT474, SK-BR-3, MCF-7 naive, MDA-MB-231, HEK 293, CHO-p185, CHO-p110, CHO-p95, U-251 + TurboGFP, MDA-MB-231 GFP Actin, MDA-MB-231 -sh scramble GFP Actin, MDA-MB-231 GFP Actin+HSF1 OE, MDA-MB-231 GFP Actin +RFP lenti, MDA-MB-231 GFP Actin+sh HSF1B, MDA-MB-231 GFP control, BJ fibroblasts, U251 naïve, HeLa
Date tested: 10/31/19
Client: Alisha Geldert

Mycoplasma test: NEGATIVE 10/31/19: Cells were fixed with methanol and stained with Hoechst nuclear stain to visualize nuclei. Small nuclei present in the cellular membrane indicate mycoplasma infection.

Disclaimer: While every reasonable effort has been made to assure the accuracy of these data, no warranty, express or implied, is made by this facility.

Alison Killilea

Alison N. Killilea, Ph.D. | Bioscience Facility Manager
